# Interferon Regulatory Factor 5 Mediates Lipopolysaccharide-Induced Neuroinflammation

**DOI:** 10.3389/fimmu.2020.600479

**Published:** 2020-12-09

**Authors:** Ziqi Fan, Shuai Zhao, Yueli Zhu, Zheyu Li, Zhirong Liu, Yaping Yan, Jun Tian, Yanxing Chen, Baorong Zhang

**Affiliations:** ^1^ Department of Neurology, The Second Affiliated Hospital, School of Medicine, Zhejiang University, Hangzhou, China; ^2^ Department of Geriatrics, The First Affiliated Hospital, School of Medicine, Zhejiang University, Hangzhou, China

**Keywords:** IRF5, siRNA interfering, intracerebroventricular, lipopolysaccharide, neuroinflammation

## Abstract

**Background:**

Activated microglia play a vital role in neuroinflammation in the central nervous system (CNS), which is associated with the pathogenesis and the progression of neurological diseases. Interferon regulatory factor 5 (IRF5) has been well established participating in inflammatory responses and is highly expressed in M1 macrophage in the periphery, the role of which in the CNS remains elusive.

**Methods:**

Lipopolysaccharide (LPS) was employed to induce neuroinflammation. Down-regulation of IRF5 in C57/BL6 mice and BV2 microglial cells were achieved by IRF5 siRNA transfection. The levels of pro-inflammatory cytokines were evaluated by ELISA and quantitative real-time PCR. The expression levels of IRF5 were examined by immunofluorescence and Western blot.

**Results:**

LPS induced significantly elevated expression of IRF5 in mouse brain, which co-localized with CD11b-positive microglia. Down-regulation of IRF5 quenched the pro-inflammatory responses. The levels of pro-inflammatory cytokines TNF-α, IL-1β, and IL-6 were up-regulated at 4 h after LPS treatment, which were significantly down-regulated with the knockdown of IRF5. LPS-induced pro-inflammatory responses were transient, which were comparable to control group at 24 h after LPS treatment. However, LPS did not up-regulate the expression of IRF5 in BV2 microglial cells, indicating that LPS-induced inflammation in BV2 cells does not involve IRF5 signaling.

**Conclusions:**

IRF5 mediates the inflammatory responses in the CNS, which might serve as a therapeutic target for CNS inflammatory diseases. LPS-induced inflammation does not involve IRF5 signaling in BV2 microglia.

## Introduction

Microglia play a central role in the immune surveillance of the central nervous system (CNS), which is activated under pathological conditions to maintain the homeostasis of CNS (1). The profile of microglia is consistent with macrophages, which are differentiated from inflammatory monocytes (2, 3).

The interferon regulatory factor (IRFs) family with nine members (IRF1, IRF2, IRF3, IRF4/Pip/ICSAT, IRF5, IRF6, IRF7, ICSBP/IRF8, and ISGF3g/p48/IRF9) are known to play a central role in both innate and adaptive immune responses, and cell development including dendritic cells (DCs), NK cells, T cells, B cells, and myeloid cells (4–6). Studies in the peripheral indicate that IRFs are integral components of the macrophage activation (4). As reported, IRF1, IRF5, and IRF8 are critical for M1 (pro-inflammatory) macrophage polarization, the expression of which would be upregulated under inflammatory responses (7–9). IRF2 and IRF4 are essential for anti-inflammatory responses and IRF4 is associated with M2 macrophage polarization (10). IRF3, IRF7, and IRF9 were initially found in virus infection and play a critical role in the immune system. In addition, IRF3 and IRF7 have been well characterized in toll like receptor (TLR) mediated inflammation responses. As particular, IRF5 is a crucial regulator of the cell cycle, apoptosis, and inflammation in the peripheral. It is also required for the expression of TLR (TLR4/7/8/9) mediated pro-inflammatory cytokines like TNF, IL-6, IL-12, and so on (4, 11). In human, *IRF5* gene polymorphisms are associated with several inflammatory and autoimmune diseases including systemic lupus erythematosus (SLE), inflammatory bowel disease, and rheumatoid arthritis (6). However, the role of IRF5 in the CNS remains elusive. A recent study found that the expression of IRF5 influenced stroke outcomes (12), which was related to IRF5 mediated activation of microglia and regulation of neuroinflammation. Thus, our study intends to clarify the role of IRF5 in microglia-associated neuroinflammation. Given the difficulties in manipulating the genes in microglia because of its high immune-reactivity, we chose RNA interference to achieve the knockdown of IRF5 in microglia both *in vitro* and *in vivo*.

Lipopolysaccharide (LPS) is widely used as a positive stimulus to mimic acute inflammatory responses through activating TLR4. Pro-inflammatory cytokines like TNF-α, IL-1β, and IL-6 (13) can be up-regulated with the treatment of LPS, which exacerbate neurotoxicity (14). LPS has been employed to induce neuroinflammation and age-related progression of cognitive impairments in the investigation of inflammatory related diseases (15). Neurodegenerative diseases such as Alzheimer's disease, were also modeled with LPS by inducing neuroinflammatory responses in the brain. Therefore, in this study, we also induced neuroinflammatory responses in the CNS with intracerebroventricular (ICV) injection of LPS, and investigated the role of IRF5 in microglia-mediated neuroinflammatory responses.

## Materials and Methods

### Cell Culture and Small Interfering RNA Transfection

BV2 microglial cells were seeded in 6-well plates with appropriate density (1.0 × 10^6^/well) before transfection, which were maintained in humidified incubators at 37°C in a 5% CO_2_ incubator for about 24 h. In the RNA interfering test, siRNA and shRNA, assigned by Genephrama, Shanghai, China, were transfected with lipofectamine 3000 for 24 h. LPS (O111:B4, Sigma, USA) solution (diluted in cell culture medium) were added into wells containing cells for 4 or 24 h before collection. Sequences of the IRF5 siRNA, the non-targeting scramble siRNA, and shRNA used in this study were as follows.

IRF5:siRNA-1 sense sequence: 5′-GCAGUUUAAAGAGCUUCAUUU-3′

antisense sequence: 5′-AUGAAGCUCUUUAAACUGCUU-3′

siRNA-2 sense sequence: 5′-GCCUAGAGCAGUUUCUCAAUU-3′

antisense sequence: 5′-UUGAGAAACUGCUCUAGGCUU-3′

Scramble siRNA: sense sequence: 5′-GUUAGAAAGGGCAGAUAAAUU-3′

antisense sequence: 5′-UUUAUCUGCCCUUUCUAACUU-3′

shRNA sequence: GATCCCCGCCTAGAGCAGTTTCTCAATGCGAACATTGAGAAACTGCT

CTAGGCTTTTTGGAAAT

### Animals

C57/BL6 mice were obtained from SLAC laboratory animal company (Shanghai, China). They were housed on a 12 h light/dark cycle with room temperature at 22°C, and had *ad libitum* access to food and water. All procedures were approved by the Institutional Animal Care and Use Committee of Zhejiang University and conducted in accordance with the National Institutes of Health guide for the care and use of laboratory animals (NIH Publication No. 85-23, revised 1996) guidelines for ethical treatment of animals.

### Intracerebroventricular (ICV) Injection of siRNA and LPS

Eleven-week-old male mice were anesthetized with intraperitoneal administration of Avertin (1.25%, 20 μl/g of body weight, Sigma, loss of toe pad reflexes), and then fixed on a stereotaxic instrument (RWD, Shenzhen, China) in a flat position. The bregma coordinates used for injection were −1.0 mm lateral, −0.3 mm posterior, and −2.5 mm below. SiRNA (IRF5 or scramble) with transfection reagent (4 μl) was injected, followed by injection of LPS (O111:B4, 2 mg/ml, 2 μl) or normal saline (NS) 24 h later. Mice were sacrificed 4 h or 24 h after LPS injection.

### Western Blot

Brain tissues including cortex and hippocampus were homogenized in homogenization buffer with protease inhibitors containing 50 mM Tris-HCl (pH 7.4), 2 mM EDTA, 2 mM EGTA, 2 mM Na_3_VO_4_, 50 mM NaF, 20 mM β-glycerophosphate, 0.5 mM AEBSF, 10 μg/ml aprotinin, 10 μg/ml leupeptin, and 4 μg/ml pepstatin A. BV2 microglial cells were lysed on ice with RIPA lysis buffer (Thermo Fisher Scientific, USA). Protein was separated by gel electrophoresis and transferred to PVDF membranes (Millipore, Bedford, MA, USA). Membranes were blocked with 5% skim milk in 0.1% Tris-buffered saline/Tween-20 (TBST) for 1 h, and then incubated with primary antibody IRF5 (1:500, Abcam) overnight at 4°C. After incubation with the appropriate HRP-conjugated secondary antibody for 1 h at room temperature, the membranes were detected by the ECL-PLUS system (ECL, Pierce, and Rockford, USA). The signal intensity was analyzed with Image Lab (BIO-RAD, Hercules, CA).

### Immunofluorescence

Mice were anesthetized by intraperitoneal injection of Avertin (0.02 ml/g) and perfused transcardially with ice-cold 4% paraformaldehyde (PFA)/PBS. The brains were fixed with 4% PFA for 2 h, followed by cryoprotection in 30% sucrose for 24 h at 4°C. Coronal sections were cut immediately after embedding. Following incubation overnight at 4 °C with primary antibodies, rat anti-CD11b (1:500, AbD Serotec) and, rabbit anti-IRF5 (1:500, Abcam), brain sections were incubated with secondary antibodies conjugated to Alexa Fluor 488 or Cy3 (1:1000, Thermo Fisher). The sections were then mounted on microscope slides after dying with DAPI (1:10000, Sigma, USA) for 10 min. For every 10 sections, one section was selected and analyzed using Nikon A1 confocal microscopy (Nikon, Japan).

### Quantitative Real-Time PCR

Total RNA was isolated with TRIZO (invitrogen, Canada). First-strand cDNA was synthesized from total RNA by using 5× Prime Script RT master mix (Takara, Japan). Secondary reacting step was real-time PCR using TB Green Premix Ex Taq II (Takara, Japan) with Step One Plus Real-Time PCR System (Applied Biosystems, Foster City, CA, USA). Gene primers for quantitative PCR below were designed from National Center for Biotechnology Information database.

IRF5: 5′-CAGGTGAACAGCTGCCAGTA-3′ (forward)

5′-GGCCTTGAAGATGGTGTTGT-3′ (reverse)

GAPDH: 5′-GTGTTCCTACCCCCAATGTGT-3′(forward)

5′-ATTGTCATACCAGGAAATGAGCTT-3′(reverse)

### Enzyme-Linked Immunosorbent Assay (ELISA)

Frozen tissue was homogenized in ice-cold buffer as mentioned above. The homogenates were diluted 1:5 before ELISA was carried out. TNF-α, IL-1β, and IL-6 were subsequently measured using ELISA kits (ABclonal, China) according to the manufacturer’s instructions. Three replicate wells were set up for each sample.

### Statistical Analysis

Data are presented as mean ± SEM, and differences among groups were determined with GraphPad Prism Version 7 by one-way ANOVA, followed by the Bonferroni *post hoc* test. PCR data were expressed as 2^(-ΔΔCT). *P*<0.05 was considered statistically significant.

## Results

### LPS-Induced Inflammation in BV2 Microglia Does Not Involve IRF5 Signaling

**Figure 1:**
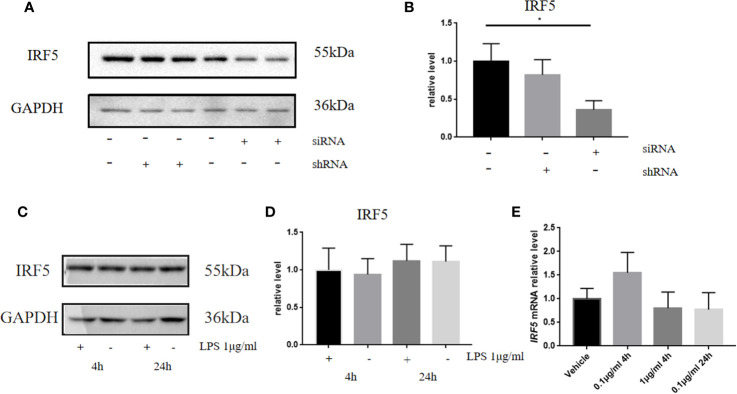
LPS failed to affect the expression of IRF5 in BV2 microglia. **(A)** The expression levels of IRF5 in BV2 microglia at 24 h after the transfection of siRNA or shRNA were determined by Western blot. **(B)** Densitometric quantification of the blots in **(A)** after being normalized with the GAPDH levels. **(C)** The expression levels of IRF5 in BV2 microglia at 4 h or 24 h after LPS (1μg/ml) challenge. **(D)** Densitometric quantification of the blots in **(C)** after being normalized with the GAPDH levels. **(E)** The relative levels of IRF5 in BV2 microglia at 4 or 24 h after LPS (0.1 or 1μg/ml) challenge were determined by quantitative real-time PCR. All data is generated from 3 to 4 experimental replicates. *P < 0.05. Values are the mean ± SEM.

**Figure 2:**
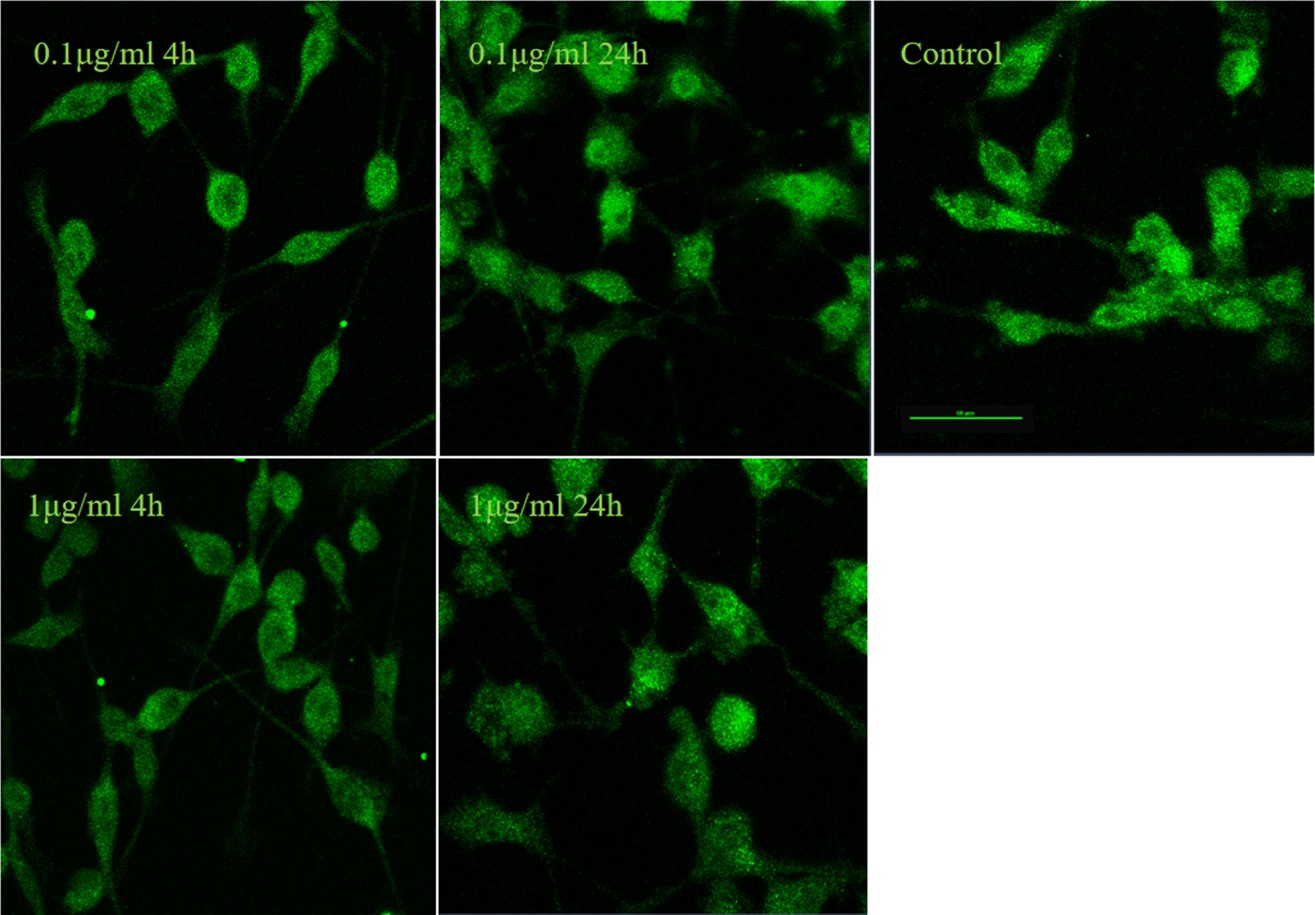
LPS failed to induce nuclear translocation of IRF5 in BV2 microglia. IRF5 (green) nuclear translocation were determined by immunofluorescence at 4 h and 24 h after LPS (0.1or 1μg/ml) treatment. Scale bar, 50μm.

It is well established that LPS could induce inflammatory responses in BV2 microglial cells. Therefore, we initially intended to investigate the role of IRF5 *in vitro* with BV2 microglial cells. Knockdown of the basal level of IRF5 in BV2 cells was achieved with IRF5 siRNA but not shRNA (**Figures 1A, B**). However, LPS (0.1 or 1 μg/ml, the commonly used concentrations for LPS challenge) did not affect the expression of IRF5 in BV2 microglial cells at 4 or 24 h (**Figures 1C–E**). Nuclear translocation of IRF5 was not observed either with the treatment of LPS at 4 h or 24 h as detected by immunofluorescence (**Figure 2**). To the contrary, LPS induced up-regulation of IRF5 expression in primary microglia at both 4 h and 24 h, which was lower at 24 h (**Supplementary Figures 1A, B**). These results indicate that LPS-induced inflammation does not involve IRF5 signaling pathway in BV2 microglia.

### LPS Induced the Activation of Microglia and Up-regulation of IRF5 in Mouse Brain

**Figure 3:**
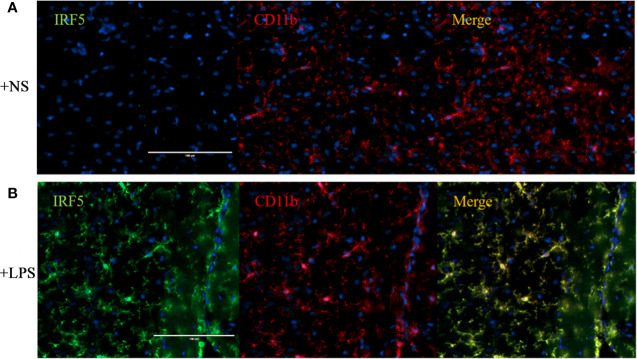
ICV injection of LPS increased the expression of IRF5 in mouse brain. Mice were treated with ICV injection of LPS. The brains were collected after 4 h of injection (n = 4 in each group). Representative images of the immunofluorescence staining of IRF5 (green) and CD11b (red, microglial marker) in the cortex of NS **(A)** and LPS **(B)** injected group are shown. Scale bar, 100μm.

To further confirm the involvement of IRF5 in LPS-induced microglia activation *in vivo*, ICV injection of LPS was employed to induce neuroinflammation in the brain. As shown in **Figure 3A**, the basal level of IRF5 in mouse brain was relatively low. LPS induced the activation of microglia, which was evidenced by the observation of CD11b-positive (CD11b^+^) microglia in ramified morphology in the cortex (**Figure 3B**). Meanwhile, IRF5 was up-regulated and co-localized with CD11b^+^ microglia after the treatment of LPS (**Figure 3B**). Thus, our data suggest that LPS could induce the activation of microglia and the up-regulation of IRF5 in mouse brain.

### Knockdown of IRF5 Alleviated LPS-Induced Neuroinflammation

**Figure 4:**
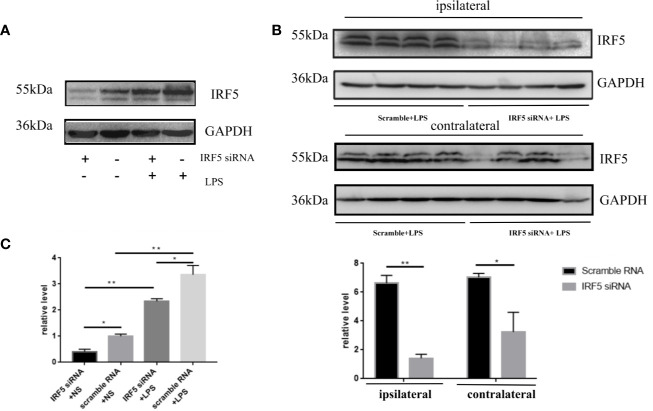
IRF5 siRNA down-regulated the increased expression of IRF5 induced by LPS. Mice were treated with ICV injection of IRF5 siRNA or scramble RNA, followed by ICV injection of LPS 24 h later. Mice were sacrificed 4 h later and the brain tissues were collected. **(A)** The expression levels of IRF5 in the ipsilateral hemispheres were determined by Western blot. **(B)** The expression levels of IRF5 in the ipsilateral and contralateral hemispheres were determined by Western blot. **(C)** Densitometric quantification of the blots in **(A, B)** after being normalized with the GAPDH levels. n = 4 in each group, *P < 0.05, **P < 0.01. Values are the mean ± SEM.

**Figure 5:**
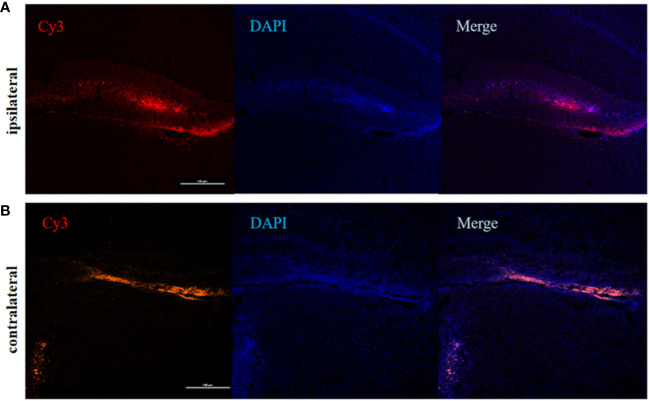
The distribution of IRF5 siRNA in the brain. Brain tissues were collected 24 h after ICV injection of Cy3-labeled siRNA (red). **(A)** Fluorescence in DG area (red) of the hippocampus in the ipsilateral **(A)** and contralateral **(B)** hemispheres (n = 4 in each group). Scale bar, 100μm.

To determine the involvement of IRF5 in LPS-induced neuroinflammation, the expression of IRF5 in mouse brain was knocked down by IRF5 siRNA. As shown in **Figure 4A**, LPS increased the expression of IRF5, which was down-regulated by IRF5 siRNA. The knockdown of IRF5 with siRNA in the ipsilateral hemisphere has less variation than the contralateral hemisphere (**Figures 4B, C**). Therefore, we only used the ipsilateral brains for the following experiments. To further confirm the distribution of IRF5 siRNA in the brain following ICV injection, we used Cy3-labled IRF5 siRNA and found that siRNA was widely distributed in the ipsilateral hippocampal DG area at 24 h after transfection, which was much less in the contralateral hippocampus (**Figure 5**). No immunofluorescence was observed in other area of the brain such as the ipsilateral or contralateral cortex.

**Figure 6:**
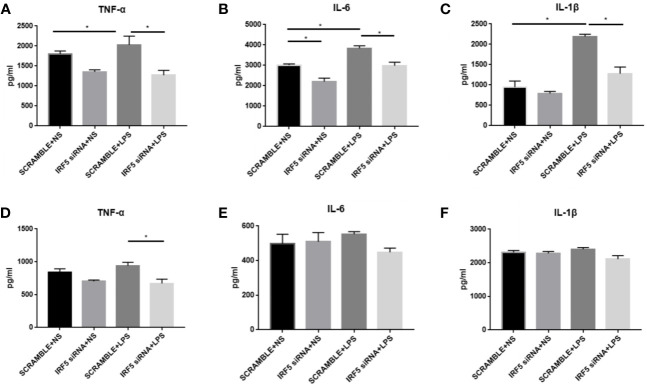
LPS-induced increased synthesis of pro-inflammatory responses were attenuated after down-regulation of IRF5. Mice were treated with ICV injection of IRF5 siRNA or scramble RNA, followed by ICV injection of LPS 24 h later. **(A–C)** Mice were sacrificed at 4 h after LPS treatment and the brain tissues were collected. The levels of TNF-α, IL-6, and IL-1β were detected by ELISA. **(D–F)** Mice were sacrificed at 24 h after LPS treatment and the brain tissues were collected. The levels of TNF-α, IL-6, and IL-1β were detected by ELISA. n = 4–6 in each group, *P<0.05. Values are the mean ± SEM.

To evaluate the inflammatory responses, the levels of pro-inflammatory cytokines TNF-α, IL-1β, and IL-6 were examined at both 4 and 24 h after LPS treatment. LPS significantly up-regulated the synthesis of TNF-α, IL-1β, and IL-6 at 4 h after treatment (**Figures 6A–C**). However, this phenomenon was transient. No significant increase in these pro-inflammatory factors were observed in LPS treated mice as compared to those in NS treated mice at 24 h after treatment (**Figures 6D, F**). Knockdown of IRF5 by siRNA significantly reduced the synthesis of TNF-α, IL-1β, and IL-6 at 4 h after LPS treatment (**Figure 6A**). These results suggest a rapid response to LPS with the induction of pro-inflammatory cytokines, which is mediated through IRF5.

## Discussion

In the present study, we showed for the first time that IRF5 was involved in LPS-induced acute neuroinflammatory responses in the CNS. Down-regulation of IRF5 could alleviate the synthesis of pro-inflammatory cytokines such as TNF-α, IL-1β, and IL-6. SiRNA interfered with the transcription of IRF5 and inhibited the synthesis of pro-inflammatory cytokines. The technique employed in this study by using siRNA with lipid vector *in vivo* is innovative and feasible, which could be used for our further studies.

Microglia, the innate immune cells of the CNS, play a central role in neural injury and repair in the CNS. Initiation of acute inflammatory responses, with the release of excessive pro-inflammatory cytokines such as TNF-α, IL-1β, and IL-6, can induce neuronal damage and synaptic dysfunction (13, 16, 17). Microglia mediated inflammation is involved in the development of numerous neurodegenerative disease such as Alzheimer’s disease, demyelinative diseases like multiple sclerosis, and other neurological diseases like ischemic stroke (18). Thus, controlling the induction of pro-inflammatory responses by microglia would be beneficial for neuroinflammatory diseases (19).

IRF5 is mainly expressed in macrophages, and plays a central role in the innate immune responses (20, 21). It has been reported to be the main regulator of the induction of pro-inflammatory cytokines expression, promoting the M1 macrophage polarization in the periphery, and performing as a downstream regulator of the TLR-MyD88 signaling pathway (11). IRF5 has also been shown to be responsible for microglia mediated neuropathic pain after peripheral nerve injury (PNI) (7), the expression of which was up-regulated in microglia after PNI. In the current study, we also found increased expression of IRF5 in microglia with the treatment of LPS, indicating the involvement of IRF5 in LPS-induced neuroinflammation. Besides, knockdown of IRF5 by siRNA significantly reduced the LPS-induced release of pro-inflammatory cytokines TNF-α, IL-1β, and IL-6. These results suggest that targeting the expression of IRF5 in microglia could attenuate the pro-inflammatory activation of microglia induced by LPS. It is known that microglia have two activation states after pathogenic stimulation, namely pro-inflammatory (M1) and anti-inflammatory (M2) phenotypes. M1 microglia initially respond to the pathological stimulus and promote the destruction of invading pathogens, which is accompanied by the release of pro-inflammatory cytokines, inducing neurotoxicity and acute inflammation (22). M2 microglia, on the other hand, can produce anti-inflammatory cytokines such as IL-4 and IL-10, which could dampen the pro-inflammatory responses and promote the resolution of inflammation and tissue repair (23, 24). Actually, these pro- and anti-inflammatory responses are not “all or none” pattern. Manipulating the polarization of microglia towards the M2 phenotype might attenuate neuroinflammation related brain injuries (12, 25). A recently study showed that down-regulation of IRF5 in transient middle cerebral artery occlusion mice attenuated M1, but enhanced M2 activation of microglia, quenched pro-inflammatory responses, and improved stroke outcomes (12, 26). Therefore, IRF5 mediated pro-inflammatory activation of microglia are not disease specific, but can also be seen in other pathogenic conditions. In our study, we found that the levels of pro-inflammatory cytokines IL-1β and IL-6 returned to baseline by 24 h after LPS treatment. This is consistent with a previous study, which also demonstrated a transiently (< 24 h) increased expression of pro-inflammatory factors following LPS treatment. In addition, the level of TNF-α was still lower in IRF5 knockdown group by 24 h after LPS treatment, indicating a prolonged inhibition of TNF-α expression.

BV2 microglia are the most frequently used cell line as an alternative to primary microglia because it was originally derived from v-raf/v-myc-immortalized murine neonatal microglia (27). It is widely used as an *in vitro* model system to investigate neuroinflammation, especially involving microglial TLR signaling pathway (28). Therefore, we also used this cell line initially to investigate the role of IRF5 in LPS-induced neuroinflammation. However, we failed to observe any changes in the expression of IRF5 following LPS treatment. This is not due to insufficient LPS concentration used. Different LPS concentrations (0.01–1 μg/ml) have been used for inducing pro-inflammatory responses in BV2 cells in previous studies, which invariably showed increased expression of pro-inflammatory cytokines (7, 13). Besides, prolonged treatment of LPS also failed to induce increased expression of IRF5. These observations indicate that LPS-induced neuroinflammation in BV2 microglia is not mediated by IRF5. Similarly, in a RNA sequencing study, it was shown that LPS-induced expression profile of IRFs in primary microglia was very much different from that in BV2 microglia. Induction of IRF5 expression was only observed in LPS stimulated primary microglia, not BV2 microglia (24, 29). However, amyloid β successfully induced the expression of IRF5 and the subsequent pro-inflammatory cytokines in BV2 microglia (data not shown), suggesting differential signaling pathways involved in LPS and amyloid β induced activation of BV2 microglia. On the other hand, significantly increased expression of IRF5 and pro-inflammatory cytokines in mouse brain following ICV injection of LPS was observed in our study, which could be reversed with the knockdown of IRF5. These results together suggest that BV2 microglial cells could not completely substitute primary microglia *in vitro* and is not an ideal cell line for studies involving IRF5 mediated TLR signaling pathway. The mechanism underlying the differential responses of BV2 and primary microglia to LPS challenge is still elusive. It is known that LPS can activate a series of intracellular inflammatory signaling pathways in microglia (24, 29), like the TLR4-mediated Transforming Growth Factor-Beta-Activated Kinase 1/I-Kappa-B Kinase/Nuclear Factor of Kappa Light Polypeptide Gene Enhancer in B-Cells (TAK1/IKK/NF-κB), mitogen-activated protein kinases (MAPK), and Akt signaling pathways, and consequently, increase the expression of pro-inflammatory cytokines. However, the activation of IRF5 vary with the cell type and stimulation. As shown in the present study, amyloid β but not LPS could induce the activation of IRF5 in BV2 microglia. Tumor necrosis factor receptor-associated factors 6 (TRAF6) and receptor-interacting protein kinase 2 (RIP2) have been implicated in the activation of IRF5 (11, 30). Although BV2 cell lines exhibit high similarity to primary microglial cells, the latter reveal a unique molecular expression pattern under different conditions. Therefore, it is possible that unlike in primary microglia, LPS-induced production of pro-inflammatory cytokines in BV2 cell line involves signaling pathways independent of RIP2/TRAF6-mediated IRF5 activation. Although it was not feasible to activate IRF5 in BV2 microglia following LPS, the basal level of IRF5 was detectable, which could still be used for assessing the efficiency of IRF5 knockdown by siRNA.

Here we characterized the RNA interfere (RNAi) technique to regulate the expression of IRF5 successfully both *in vitro* and *in vivo*. Genetic manipulation with siRNA was frequently used to investigate the role of a specific protein by stimulating the complementary target mRNA silencing (31, 32). Though viral vectors have been established for high efficiency of nucleic acid delivery, they are also at high risk of up-regulating pro-inflammatory factors. After trying several kinds of vectors and transfection reagents, we found that lipid-binding system with methylated siRNA could achieve high transfection efficiency with low pro-inflammatory responses, which can be used successfully for *in vivo* experiments.

## Conclusions

In conclusion, we demonstrated that like in the periphery, IRF5 also plays an essential role in the activation of microglia in the CNS. IRF5 mediated LPS-induced pro-inflammatory activation of microglia, down-regulation of which could attenuate the pro-inflammatory responses. Targeting IRF5 could be a promising therapeutic for the treatment of various CNS inflammatory diseases. However, further studies are undoubtedly needed to confirm the involvement of IRF5 in neuroinflammation under different pathogenic conditions. In addition, BV2 microglia is not an ideal cell line for investigating IRF5 mediated neuroinflammation under LPS challenge.

## Data Availability Statement

The original contributions presented in the study are included in the article/**Supplementary Material**. Further inquiries can be directed to the corresponding authors.

## Ethics Statement

The animal study was reviewed and approved by Institutional Animal Care and Use Committee of Zhejiang University.

## Author Contributions

ZF, SZ, and YC participated in the design of the study. ZF and SZ performed the experiments. YZ, ZL, JT, and YY participated in the maintenance of the animal colony, sample collection, and data analysis. YC and BZ gave the pivotal answers and guidance to the experiment and manuscript revision. All authors read and approved the final manuscript. All authors contributed to the article and approved the submitted version.

## Funding

This study was supported by the National Natural Science Foundation of China (81520108010 and 81870826), Zhejiang Provincial Natural Science Foundation of China (LY18H090004 and LY17H090003) and Guangdong Provincial Key S&T Program (2018B030336001).

## Conflict of Interest

The authors declare that the research was conducted in the absence of any commercial or financial relationships that could be construed as a potential conflict of interest.
