# Supplementary figures and images for: Interferon Regulatory Factor 5 Mediates Lipopolysaccharide-Induced Neuroinflammation
Source: Front Immunol. 2020 Dec 9;11:600479. doi: 10.3389/fimmu.2020.600479 (PMC7755991; doi:10.3389/fimmu.2020.600479)

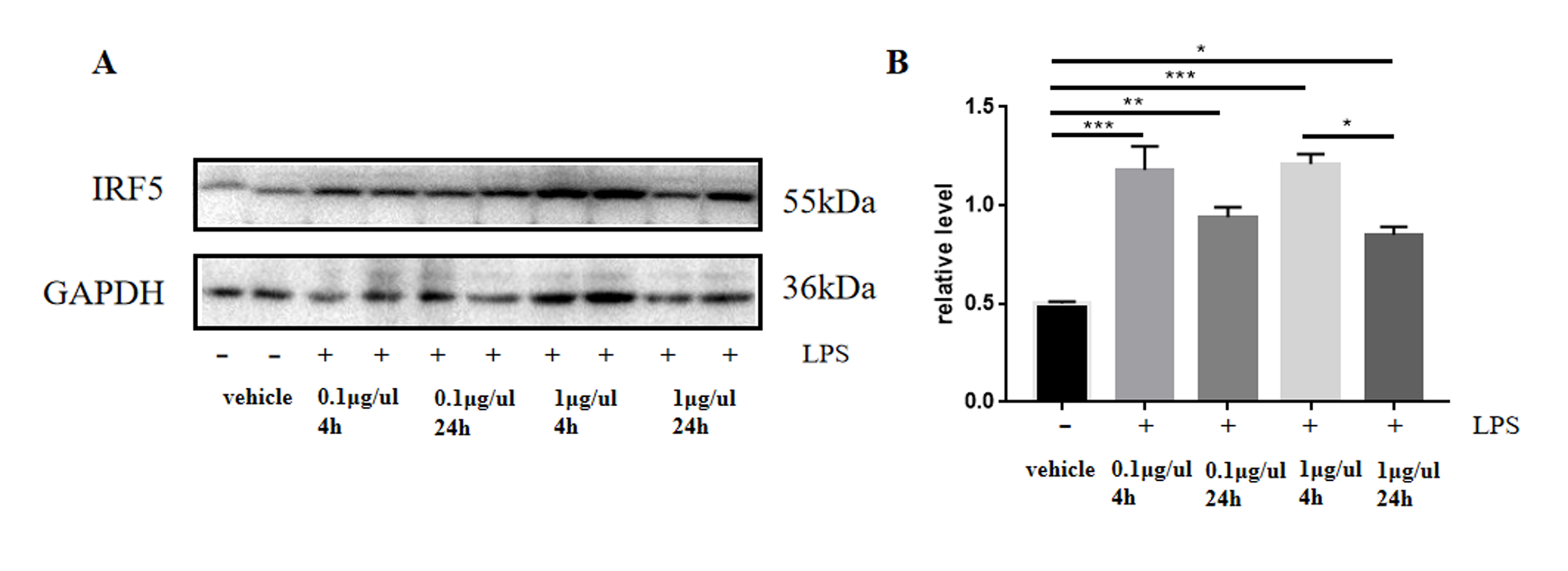

Supplement: Supplementary file 1 [file Image_1.tif]
