# Supplementary material for: Interferon Regulatory Factor 5 Mediates Lipopolysaccharide-Induced Neuroinflammation
Source: Front Immunol. 2020 Dec 9;11:600479. doi: 10.3389/fimmu.2020.600479 (PMC7755991; doi:10.3389/fimmu.2020.600479)
Supplement: Supplementary file 2 [file Presentation_1.pdf]

Supplementary method

Primary microglia culture

Brain was removed from P0 SD rat (SLAC laboratory animal company, Shanghai, China) in ice-chilled dishes. After removing the meninges, the cerebral cortex was dissected and dissociated in Hank's balanced salt solution (HBSS). After trypsinization and centrifugation, the cells were plated on poly-D-lysine-coated T75 flasks and maintained in Dulbecco's modified eagle medium (DMEM) containing 10% FBS (GIBICO, USA) with antibiotics for 7 to 8 days. The flasks were then agitated on an orbital shaker (200 rpm, at 37 °C) for 2 h. Cell suspension was collected and seeded into 6-well plates. Cell were incubated for 1 h to enable microglia to attach to the dish, and then washed once to remove non-adherent cells (astrocytes and oligodendrocytes) for further LPS treatment. The purity of the microglia (over 95%) was determined by immunofluorescence analysis using mouse anti-Iba1 (1:500, Abcam, USA).
